# *N*-(2-Nitro­phenyl­sulfon­yl)-*N*-(4-nitro­phenyl­sulfon­yl)methyl­amine

**DOI:** 10.1107/S1600536808027499

**Published:** 2008-09-06

**Authors:** Haiyan Lu

**Affiliations:** a The Graduate School of the Chinese Academy of Sciences, Beijing 100049, People’s Republic of China

## Abstract

In the crystal structure of the title compound, C_13_H_11_N_3_O_8_S_2_, mol­ecules are linked by inter­molecular C—H⋯O hydrogen bonds into zigzag chains running parallel to the *c* axis. Centrosymmetrically related chains are further stabilized by aromatic π–π stacking inter­actions [centroid–centroid distance = 3.749 (3) Å] involving adjacent 4-nitro­benzene rings. Intra­molecular C—H⋯O hydrogen bonds are also present.

## Related literature

For the crystal structures of related compounds, see: Henschel *et al.* (1996 ▶); Curtis & Pavkovic (1983 ▶). For details of the biological activities of sulfonamide compounds, see: Kamoshita *et al.* (1987 ▶). For details of the application of sulfonimade catalysts, see: Zhang *et al.* (2007 ▶). For bond-length data, see: Allen *et al.* (1987 ▶).
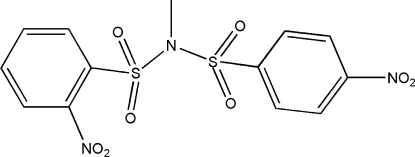


## Experimental

### 

#### Crystal data

C_13_H_11_N_3_O_8_S_2_
*M* *_r_* = 401.37
Monoclinic, 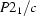

*a* = 13.517 (3) Å
*b* = 9.994 (2) Å
*c* = 11.990 (2) Å
β = 95.26 (3)°
*V* = 1613.0 (6) Å^3^
*Z* = 4
Mo *K*α radiation
μ = 0.38 mm^−1^
*T* = 153 (2) K
0.58 × 0.47 × 0.29 mm

#### Data collection

Rigaku R-AXIS RAPID IP area-detector diffractometer
Absorption correction: multi-scan (*ABSCOR*; Higashi 1995 ▶) *T*_min_ = 0.750, *T*_max_ = 0.897
15376 measured reflections
3683 independent reflections
3540 reflections with *I* > 2σ(*I*)
*R*_int_ = 0.017

#### Refinement

*R*[*F* ^2^ > 2σ(*F* ^2^)] = 0.030
*wR*(*F* ^2^) = 0.094
*S* = 1.13
3683 reflections
236 parameters
H-atom parameters constrained
Δρ_max_ = 0.49 e Å^−3^
Δρ_min_ = −0.41 e Å^−3^

### 

Data collection: *RAPID-AUTO* (Rigaku, 2004 ▶); cell refinement: *RAPID-AUTO*; data reduction: *RAPID-AUTO*; program(s) used to solve structure: *SHELXTL* (Sheldrick, 2008 ▶); program(s) used to refine structure: *SHELXTL*; molecular graphics: *SHELXTL*; software used to prepare material for publication: *SHELXTL*.

## Supplementary Material

Crystal structure: contains datablocks I, global. DOI: 10.1107/S1600536808027499/rz2239sup1.cif

Structure factors: contains datablocks I. DOI: 10.1107/S1600536808027499/rz2239Isup2.hkl

Additional supplementary materials:  crystallographic information; 3D view; checkCIF report

## Figures and Tables

**Table 1:** Hydrogen-bond geometry (Å, °)

*D*—H⋯*A*	*D*—H	H⋯*A*	*D*⋯*A*	*D*—H⋯*A*
C8—H8*B*⋯O7	0.95	2.53	2.902 (2)	104
C4—H4*A*⋯O5	0.95	2.38	2.803 (2)	106
C13—H13*A*⋯O7	0.98	2.54	2.978 (2)	107
C13—H13*C*⋯O1	0.98	2.34	2.972 (2)	122
C1—H1*A*⋯O6^i^	0.95	2.51	3.369 (2)	150

Symmetry code: (i) 
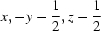
.

**Fig. 1.**
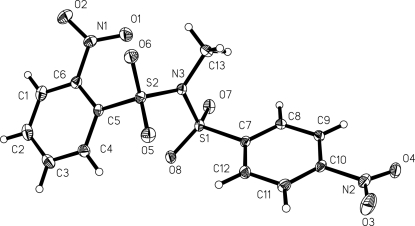
The molecular structure of the title compound, with the atom-labelling scheme. Displacement ellipsoids are drawn at the 40% probability level.

**Fig. 2.**
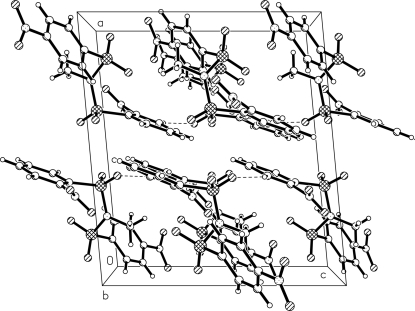
Packing diagram of the title compound viewed down the b axis, showing the chains of molecules formed by intermolecular hydrogen bonds (dashed lines).

## supplementary crystallographic information

### Comment

Molecules containing the sulfonimide group have been recently of interest for their applications as herbicides (Kamoshita *et al.*,  1987) and catalysts (Zhang *et al.*,  2007). In the present paper, the crystal structure of a new compound containing two sulfonimide groups is reported.

In the molecule of the title compound (Fig. 1) all bond lengths are normal (Allen *et al.*,  1987) and in a good agreement with those reported previously for similar compounds (Henschel *et al.*,  1996; Curtis & Pavkovic, 1983). The molecular conformation is stabilized by intramolecular C—H···O hydrogen bonds (Table 1). In the crystal structure, molecules are linked by intermolecular C—H···O hydrogen bonding interactions (Fig. 2) forming zigzag chains running parallel to the *c* axis. Centrosymmetrically related chains are further stabilized by aromatic π-π stacking interactions occurring between adjacent the 4-nitrobenzene rings with a centroid-centroid distance of 3.749 (3) Å.

### Experimental

A solution of 4-nitro-benzene-1-sulfonyl chloride (10 mmol, 2.21 g) in anhydrous CH_2_Cl_2_ (10 mL) was dropwise added over a period of 10 min to a solution of 2-nitro-N-methyl-benzenesulfonamide (10 mmol, 2.16 g) and EtN(i-Pr)_2_ (3 mmol) in CH_2_Cl_2_ (10 mL) at 273K. The mixture was stirred at room temperature for 4 h. The organic phase was washed with 2N HCl twice and dried over anhydrous Na_2_SO_4_. The solvent was removed and the residue was purified by flash chromatography (2:1 cyclohexane/dichloromethane) to give the title compound as a white solid (2.81 mg, 70% yield).  Single crystals suitable for X-ray measurements were obtained by slow evaporation of an ethanol/dichloromethane solution (1:1 v/v) at room temperature.

### Refinement

H atoms were positioned geometrically and refined using a riding model, with C—H = 0.95 or 0.98 Å and with *U*_iso_(H) = 1.2 (1.5 for methyl groups) times *U*_eq_(C).

### Crystal data

**Table:**

C_13_H_11_N_3_O_8_S_2_	*F*(000) = 824
*M**_r_* = 401.37	*D*_x_ = 1.653 Mg m^−^^3^
Monoclinic, *P*2_1_/*c*	Mo *K*α radiation, λ = 0.71073 Å
Hall symbol: -P 2ybc	Cell parameters from 10014 reflections
*a* = 13.517 (3) Å	θ = 6.2–55.0°
*b* = 9.994 (2) Å	µ = 0.38 mm^−^^1^
*c* = 11.990 (2) Å	*T* = 153 K
β = 95.26 (3)°	Block, colourless
*V* = 1613.0 (6) Å^3^	0.58 × 0.47 × 0.29 mm
*Z* = 4	

### Data collection

**Table:**

Rigaku R-AXIS RAPID IP area-detector diffractometer	3683 independent reflections
Radiation source: Rotating Anode	3540 reflections with *I* > 2σ(*I*)
graphite	*R*_int_ = 0.017
ω oscillation scans	θ_max_ = 27.5°, θ_min_ = 3.0°
Absorption correction: multi-scan (ABSCOR; Higashi 1995)	*h* = −17→17
*T*_min_ = 0.750, *T*_max_ = 0.897	*k* = −12→12
15376 measured reflections	*l* = −15→13

### Refinement

**Table:**

Refinement on *F*^2^	Secondary atom site location: difference Fourier map
Least-squares matrix: full	Hydrogen site location: inferred from neighbouring sites
*R*[*F*^2^ > 2σ(*F*^2^)] = 0.030	H-atom parameters constrained
*wR*(*F*^2^) = 0.094	*w* = 1/[σ^2^(*F*_o_^2^) + (0.0558*P*)^2^ + 0.7411*P*] where *P* = (*F*_o_^2^ + 2*F*_c_^2^)/3
*S* = 1.13	(Δ/σ)_max_ < 0.001
3683 reflections	Δρ_max_ = 0.49 e Å^−^^3^
236 parameters	Δρ_min_ = −0.41 e Å^−^^3^
0 restraints	Extinction correction: SHELXTL (Sheldrick, 2001), Fc^*^=kFc[1+0.001xFc^2^λ^3^/sin(2θ)]^-1/4^
Primary atom site location: structure-invariant direct methods	Extinction coefficient: 0.0231 (18)

### Special details

**Table:**

Geometry. All esds (except the esd in the dihedral angle between two l.s. planes) are estimated using the full covariance matrix. The cell esds are taken into account individually in the estimation of esds in distances, angles and torsion angles; correlations between esds in cell parameters are only used when they are defined by crystal symmetry. An approximate (isotropic) treatment of cell esds is used for estimating esds involving l.s. planes.
Refinement. Refinement of F^2^ against ALL reflections. The weighted R-factor wR and goodness of fit S are based on F^2^, conventional R-factors R are based on F, with F set to zero for negative F^2^. The threshold expression of F^2^ > 2sigma(F^2^) is used only for calculating R-factors(gt) etc. and is not relevant to the choice of reflections for refinement. R-factors based on F^2^ are statistically about twice as large as those based on F, and R- factors based on ALL data will be even larger.

### Fractional atomic coordinates and isotropic or equivalent isotropic displacement parameters (Å^2^)

**Table:**

	*x*	*y*	*z*	*U*_iso_*/*U*_eq_	
S1	0.15312 (2)	0.15728 (3)	0.41897 (3)	0.01644 (11)	
S2	0.35222 (2)	0.06267 (3)	0.48921 (3)	0.01728 (11)	
O8	0.20741 (8)	0.19778 (11)	0.32804 (8)	0.0220 (2)	
O7	0.06390 (8)	0.08053 (11)	0.39952 (9)	0.0241 (2)	
O3	0.13493 (10)	0.70583 (13)	0.72576 (12)	0.0399 (3)	
O6	0.39404 (8)	−0.02832 (12)	0.57283 (9)	0.0261 (2)	
O5	0.38943 (8)	0.19667 (11)	0.48792 (9)	0.0255 (2)	
O4	−0.01529 (9)	0.63329 (11)	0.73325 (9)	0.0271 (2)	
N3	0.23085 (9)	0.06608 (12)	0.50391 (10)	0.0187 (2)	
C7	0.12748 (10)	0.29709 (13)	0.50127 (11)	0.0161 (3)	
O2	0.34613 (11)	−0.36035 (12)	0.39097 (13)	0.0401 (3)	
C8	0.04276 (10)	0.29488 (13)	0.55885 (11)	0.0173 (3)	
H8B	−0.0011	0.2205	0.5523	0.021*	
N1	0.31903 (10)	−0.24388 (12)	0.39825 (11)	0.0241 (3)	
C6	0.36282 (10)	−0.14363 (14)	0.32761 (12)	0.0199 (3)	
C11	0.17430 (11)	0.51236 (14)	0.57559 (13)	0.0215 (3)	
H11A	0.2180	0.5870	0.5826	0.026*	
C12	0.19373 (10)	0.40442 (15)	0.50825 (12)	0.0209 (3)	
H12A	0.2509	0.4036	0.4677	0.025*	
C4	0.40219 (10)	0.08068 (15)	0.27685 (13)	0.0218 (3)	
H4A	0.4043	0.1739	0.2922	0.026*	
C10	0.08951 (10)	0.50856 (13)	0.63249 (11)	0.0177 (3)	
C5	0.36741 (10)	−0.00693 (14)	0.35469 (11)	0.0174 (3)	
O1	0.25661 (11)	−0.20743 (12)	0.45856 (12)	0.0375 (3)	
C9	0.02324 (10)	0.40325 (14)	0.62611 (11)	0.0181 (3)	
H9A	−0.0340	0.4048	0.6665	0.022*	
C3	0.43385 (11)	0.03367 (18)	0.17699 (13)	0.0269 (3)	
H3A	0.4561	0.0949	0.1241	0.032*	
N2	0.06833 (10)	0.62403 (12)	0.70259 (10)	0.0227 (3)	
C2	0.43303 (12)	−0.10224 (18)	0.15451 (13)	0.0286 (3)	
H2A	0.4578	−0.1345	0.0879	0.034*	
C13	0.19287 (12)	0.00944 (17)	0.60575 (13)	0.0268 (3)	
H13A	0.1205	0.0196	0.6011	0.040*	
H13B	0.2229	0.0569	0.6719	0.040*	
H13C	0.2100	−0.0857	0.6117	0.040*	
C1	0.39611 (11)	−0.19141 (16)	0.22913 (13)	0.0258 (3)	
H1A	0.3937	−0.2844	0.2129	0.031*	

### Atomic displacement parameters (Å^2^)

**Table:**

	*U*^11^	*U*^22^	*U*^33^	*U*^12^	*U*^13^	*U*^23^
S1	0.01570 (18)	0.01769 (18)	0.01585 (18)	0.00236 (11)	0.00106 (12)	−0.00261 (11)
S2	0.01591 (17)	0.01766 (18)	0.01801 (18)	0.00156 (11)	0.00017 (12)	−0.00121 (11)
O8	0.0226 (5)	0.0266 (5)	0.0172 (5)	0.0065 (4)	0.0044 (4)	0.0012 (4)
O7	0.0198 (5)	0.0260 (5)	0.0260 (5)	−0.0025 (4)	0.0002 (4)	−0.0080 (4)
O3	0.0363 (7)	0.0304 (6)	0.0522 (8)	−0.0013 (5)	−0.0003 (6)	−0.0206 (6)
O6	0.0242 (5)	0.0314 (6)	0.0221 (5)	0.0076 (4)	−0.0012 (4)	0.0041 (4)
O5	0.0255 (5)	0.0213 (5)	0.0295 (6)	−0.0048 (4)	0.0017 (4)	−0.0063 (4)
O4	0.0322 (6)	0.0265 (5)	0.0234 (5)	0.0113 (4)	0.0059 (4)	−0.0020 (4)
N3	0.0179 (5)	0.0196 (6)	0.0193 (6)	0.0046 (4)	0.0050 (4)	0.0025 (4)
C7	0.0167 (6)	0.0158 (6)	0.0156 (6)	0.0036 (5)	0.0007 (5)	−0.0003 (5)
O2	0.0495 (8)	0.0169 (5)	0.0556 (8)	0.0060 (5)	0.0141 (6)	0.0020 (5)
C8	0.0185 (6)	0.0170 (6)	0.0167 (6)	0.0008 (5)	0.0020 (5)	0.0019 (5)
N1	0.0279 (6)	0.0172 (6)	0.0270 (6)	−0.0001 (5)	0.0015 (5)	−0.0007 (5)
C6	0.0175 (6)	0.0200 (7)	0.0220 (7)	0.0020 (5)	0.0012 (5)	−0.0002 (5)
C11	0.0195 (6)	0.0173 (6)	0.0276 (7)	−0.0006 (5)	0.0010 (5)	−0.0013 (5)
C12	0.0167 (6)	0.0207 (7)	0.0260 (7)	0.0005 (5)	0.0054 (5)	−0.0011 (5)
C4	0.0162 (6)	0.0240 (7)	0.0253 (7)	−0.0002 (5)	0.0032 (5)	0.0026 (5)
C10	0.0215 (6)	0.0158 (6)	0.0154 (6)	0.0060 (5)	−0.0017 (5)	−0.0004 (5)
C5	0.0135 (6)	0.0193 (6)	0.0193 (6)	0.0021 (5)	0.0018 (5)	−0.0010 (5)
O1	0.0488 (7)	0.0227 (6)	0.0449 (7)	−0.0002 (5)	0.0257 (6)	0.0008 (5)
C9	0.0200 (6)	0.0196 (6)	0.0152 (6)	0.0038 (5)	0.0034 (5)	0.0025 (5)
C3	0.0198 (7)	0.0379 (8)	0.0233 (7)	−0.0007 (6)	0.0046 (6)	0.0046 (6)
N2	0.0296 (6)	0.0184 (6)	0.0192 (6)	0.0071 (5)	−0.0021 (5)	−0.0021 (5)
C2	0.0227 (7)	0.0422 (9)	0.0214 (7)	0.0056 (6)	0.0044 (6)	−0.0045 (6)
C13	0.0280 (7)	0.0300 (8)	0.0237 (7)	0.0051 (6)	0.0097 (6)	0.0079 (6)
C1	0.0237 (7)	0.0272 (7)	0.0263 (7)	0.0051 (6)	0.0005 (6)	−0.0076 (6)

### Geometric parameters (Å, °)

**Table:**

S1—O8	1.4275 (11)	C6—C5	1.4044 (19)
S1—O7	1.4304 (11)	C11—C12	1.387 (2)
S1—N3	1.6660 (13)	C11—C10	1.387 (2)
S1—C7	1.7633 (14)	C11—H11A	0.9500
S2—O6	1.4310 (11)	C12—H12A	0.9500
S2—O5	1.4312 (11)	C4—C3	1.390 (2)
S2—N3	1.6665 (13)	C4—C5	1.393 (2)
S2—C5	1.7855 (14)	C4—H4A	0.9500
O3—N2	1.2289 (19)	C10—C9	1.380 (2)
O4—N2	1.2236 (18)	C10—N2	1.4712 (17)
N3—C13	1.4799 (18)	C9—H9A	0.9500
C7—C8	1.3905 (19)	C3—C2	1.385 (2)
C7—C12	1.3950 (19)	C3—H3A	0.9500
O2—N1	1.2258 (17)	C2—C1	1.388 (2)
C8—C9	1.3901 (19)	C2—H2A	0.9500
C8—H8B	0.9500	C13—H13A	0.9800
N1—O1	1.2164 (18)	C13—H13B	0.9800
N1—C6	1.4709 (19)	C13—H13C	0.9800
C6—C1	1.387 (2)	C1—H1A	0.9500
			
O8—S1—O7	120.82 (7)	C7—C12—H12A	120.7
O8—S1—N3	106.41 (6)	C3—C4—C5	120.96 (14)
O7—S1—N3	106.34 (7)	C3—C4—H4A	119.5
O8—S1—C7	110.12 (7)	C5—C4—H4A	119.5
O7—S1—C7	108.07 (7)	C9—C10—C11	123.72 (13)
N3—S1—C7	103.69 (6)	C9—C10—N2	118.08 (13)
O6—S2—O5	119.05 (7)	C11—C10—N2	118.20 (13)
O6—S2—N3	105.57 (7)	C4—C5—C6	117.85 (13)
O5—S2—N3	109.41 (6)	C4—C5—S2	115.74 (11)
O6—S2—C5	108.34 (7)	C6—C5—S2	125.56 (11)
O5—S2—C5	106.57 (7)	C10—C9—C8	118.02 (13)
N3—S2—C5	107.42 (7)	C10—C9—H9A	121.0
C13—N3—S1	117.82 (10)	C8—C9—H9A	121.0
C13—N3—S2	119.92 (10)	C2—C3—C4	120.11 (15)
S1—N3—S2	121.22 (7)	C2—C3—H3A	119.9
C8—C7—C12	122.40 (12)	C4—C3—H3A	119.9
C8—C7—S1	118.58 (10)	O4—N2—O3	124.02 (13)
C12—C7—S1	119.00 (10)	O4—N2—C10	117.68 (12)
C9—C8—C7	118.98 (13)	O3—N2—C10	118.30 (13)
C9—C8—H8B	120.5	C3—C2—C1	120.10 (14)
C7—C8—H8B	120.5	C3—C2—H2A	120.0
O1—N1—O2	123.70 (14)	C1—C2—H2A	120.0
O1—N1—C6	118.43 (12)	N3—C13—H13A	109.5
O2—N1—C6	117.85 (13)	N3—C13—H13B	109.5
C1—C6—C5	121.40 (14)	H13A—C13—H13B	109.5
C1—C6—N1	115.78 (13)	N3—C13—H13C	109.5
C5—C6—N1	122.76 (13)	H13A—C13—H13C	109.5
C12—C11—C10	118.30 (13)	H13B—C13—H13C	109.5
C12—C11—H11A	120.8	C6—C1—C2	119.47 (15)
C10—C11—H11A	120.8	C6—C1—H1A	120.3
C11—C12—C7	118.57 (13)	C2—C1—H1A	120.3
C11—C12—H12A	120.7		
			
O8—S1—N3—C13	−179.35 (11)	C12—C11—C10—C9	0.1 (2)
O7—S1—N3—C13	−49.34 (12)	C12—C11—C10—N2	−179.14 (12)
C7—S1—N3—C13	64.50 (12)	C3—C4—C5—C6	−1.7 (2)
O8—S1—N3—S2	12.32 (10)	C3—C4—C5—S2	168.34 (11)
O7—S1—N3—S2	142.33 (8)	C1—C6—C5—C4	3.0 (2)
C7—S1—N3—S2	−103.84 (9)	N1—C6—C5—C4	−174.28 (13)
O6—S2—N3—C13	13.90 (13)	C1—C6—C5—S2	−165.97 (11)
O5—S2—N3—C13	−115.35 (12)	N1—C6—C5—S2	16.74 (19)
C5—S2—N3—C13	129.34 (12)	O6—S2—C5—C4	−134.24 (11)
O6—S2—N3—S1	−178.01 (8)	O5—S2—C5—C4	−5.02 (12)
O5—S2—N3—S1	52.74 (10)	N3—S2—C5—C4	112.16 (11)
C5—S2—N3—S1	−62.56 (10)	O6—S2—C5—C6	34.94 (14)
O8—S1—C7—C8	151.35 (11)	O5—S2—C5—C6	164.17 (12)
O7—S1—C7—C8	17.44 (13)	N3—S2—C5—C6	−78.65 (13)
N3—S1—C7—C8	−95.14 (12)	C11—C10—C9—C8	0.0 (2)
O8—S1—C7—C12	−30.00 (13)	N2—C10—C9—C8	179.28 (11)
O7—S1—C7—C12	−163.91 (11)	C7—C8—C9—C10	−0.02 (19)
N3—S1—C7—C12	83.51 (12)	C5—C4—C3—C2	−1.3 (2)
C12—C7—C8—C9	−0.2 (2)	C9—C10—N2—O4	−14.29 (18)
S1—C7—C8—C9	178.44 (10)	C11—C10—N2—O4	164.99 (13)
O1—N1—C6—C1	−152.05 (15)	C9—C10—N2—O3	166.50 (14)
O2—N1—C6—C1	26.4 (2)	C11—C10—N2—O3	−14.22 (19)
O1—N1—C6—C5	25.4 (2)	C4—C3—C2—C1	3.1 (2)
O2—N1—C6—C5	−156.21 (15)	C5—C6—C1—C2	−1.3 (2)
C10—C11—C12—C7	−0.3 (2)	N1—C6—C1—C2	176.21 (13)
C8—C7—C12—C11	0.3 (2)	C3—C2—C1—C6	−1.8 (2)
S1—C7—C12—C11	−178.29 (11)		

### Hydrogen-bond geometry (Å, °)

**Table:**

*D*—H···*A*	*D*—H	H···*A*	*D*···*A*	*D*—H···*A*
C8—H8B···O7	0.95	2.53	2.902 (2)	104.
C4—H4A···O5	0.95	2.38	2.803 (2)	106.
C13—H13A···O7	0.98	2.54	2.978 (2)	107.
C13—H13C···O1	0.98	2.34	2.972 (2)	122.
C1—H1A···O6^i^	0.95	2.51	3.369 (2)	150.

Symmetry codes: (i) *x*, −*y*−1/2, *z*−1/2.
